# Unlocking mRNA-driven CRISPR-Cas9 gene therapy via optimizing mRNA and the delivery vectors

**DOI:** 10.1016/j.omtn.2025.102737

**Published:** 2025-10-11

**Authors:** Shengyi Wang, Xiaoyu Xu, Tapani Viitala, Yilai Shu, Hongbo Zhang

**Affiliations:** 1 Joint Centre of Translational Medicine, The First Affiliated Hospital of Wenzhou Medical University, Wenzhou, Zhejiang 325015, China; 2 ENT Institute and Otorhinolaryngology Department of the Eye & ENT Hospital, State Key Laboratory of Medical Neurobiology and MOE Frontiers Center for Brain Science, Fudan University, Shanghai 200031, China; 3 Pharmaceutical Sciences Laboratory, Åbo Akademi University, 20520 Turku, Finland; 4 College of Chemistry and Material Science, Shandong Agricultural University, Taian 271018, China; 5 Institutes of Biomedical Sciences, Fudan University, Shanghai 200032, China; 6 NHC Key Laboratory of Hearing Medicine, Fudan University, Shanghai 200031, China; 7 Shanghai Key Laboratory of Gene Editing and Cell Therapy for Rare Diseases, Fudan University, Shanghai 200031, China; 8 State Key Laboratory of Brain Function and Disorders and MOE Frontiers Center for Brain Science, Fudan University, Shanghai 200032, China; 9 Division of Pharmaceutical Chemistry and Technology, Faculty of Pharmacy, University of Helsinki, 00014 Helsinki, Finland

**Keywords:** MT: RNA/DNA Editing, CRISPR-Cas9, gene therapy, engineering to mRNA, delivery vectors, length fragment integration technology

## Abstract

mRNA-driven CRISPR-Cas9 gene therapies have garnered widespread attention due to their ability to maintain highly efficient editing activity while preventing integration into the host cell genome. However, the widespread clinical application of mRNA-driven CRISPR-Cas9 gene therapy is limited by mRNA instability, strong immune responses, a short half-life, inefficient delivery *in vivo*, and off-target effects. This review summarizes recent advancements in efforts to enhance mRNA stability and translation efficiency, and it describes novel delivery vectors currently being used for mRNA-driven CRISPR-Cas9 therapies. Moreover, the development of novel gene editors based on CRISPR-Cas9 engineering and the development of length fragment integration technology based on prime editing tool engineering are also discussed. The discussion of these advances aims to provide a full picture of the challenges of mRNA-based CRISPR-Cas9 therapeutics for the treatment of various diseases.

## Graphical abstract

**Figure:**
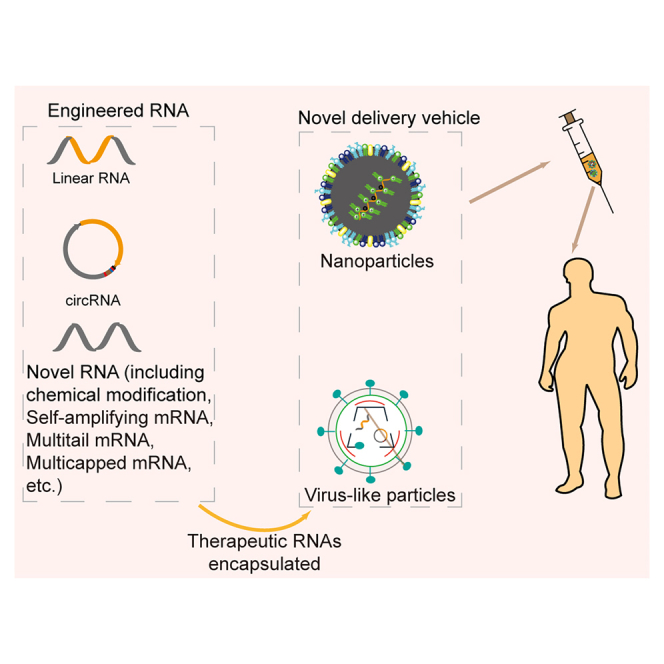

## Teaser

RNA forms of CRISPR-Cas9-based gene therapies provide more safer and effective therapeutic outcomes. This paper offers a reference for improving the effects of RNA forms of CRISPR-Cas9-based therapies, through the rational engineering design of mRNA components, combined with novel structural RNAs and novel delivery vehicles.

## Introduction

The clustered regularly interspaced short palindromic repeat (CRISPR) and associated Cas9 endonuclease (CRISPR-Cas9) system is an adaptive immune system in bacteria and archaea that has been adapted for use in genome editing.^1^^,^^2^ CRISPR-Cas9-based gene editing has revolutionized the biomedical research field and is used extensively in clinical research. However, the application of the CRISPR-Cas9 systems in clinical trials faces two major challenges: off-target effects and inefficient delivery *in vivo*.^3^^,^^4^ Significant effort has been devoted to addressing these shortcomings, and this is exemplified by the exploration of diverse forms of *in vivo* administration and the development of novel delivery vectors for CRISPR-Cas9-based gene therapy.

Three basic forms of CRISPR-Cas9, including DNA,^5^^,^^6^ mRNA,^6^^,^^7^^,^^8^ and Cas9/single-guide RNA (sgRNA) ribonucleoprotein (RNP) complexes,^6^^,^^9^ have been investigated. The DNA forms remain the most used CRISPR-Cas9-based gene therapy both *in vitro* and *in vivo*. This preference can be attributed to the more stable structure of CRISPR-Cas9 DNA, which facilitates sustained and long-term expression, thus providing enhanced editing activity.^6^^,^^10^ However, the prolonged expression of CRISPR-Cas9 DNA is also accompanied by a greater incidence of off-target events compared to the mRNA and RNP complexes.^6^^,^^11^^,^^12^ Moreover, the delivery of CRISPR-Cas9 DNA can result in unintended integration into the host genome, which can have adverse effects.^6^^,^^11^^,^^12^ Although CRISPR-Cas9 RNP complexes have the lowest rate of off-target effects among all three forms, their clinical applications are hindered by difficulties in manufacturing such complexes and by lack of efficient *in vivo* delivery vectors.^6^^,^^11^^,^^12^ CRISPR-Cas9 mRNA is considered the most promising forms for *in vivo* administration. This is because mRNA eliminates the risk of host genome integration, has a short half-life, and its action is directed toward the cytoplasm, enabling instantaneous large-scale translation without entering the nucleus, thus reducing off-target effects.^6^^,^^11^

In general, an optimal delivery vector should be designed to protect its cargo, enhance cellular internalization, facilitate endosomal escape, and target specific cells or tissues.^13^ Currently, three major vectors are employed for *in vivo* administration of the CRISPR-Cas9 systems: adeno-associated virus (AAV)-based vectors, lentivirus (LV)-based vectors, and lipid nanoparticle (LNP)-based vectors.^4^^,^^14^ AAV-based vectors are the most widely utilized and are FDA (US Food and Drug Administration) approved for gene drug delivery in clinical studies, and their extensive range of serotypes enables the infection of different cell types.^4^^,^^5^^,^^15^ However, AAV-based vectors are known to persist for years *in vivo*, which inevitably leads to greater off-target effects due to their continuous expression. Furthermore, AAV-based vectors do not directly deliver mRNA but instead use DNA as the cargo, and DNA carries the risk of integration into the host genome.^16^^,^^17^^,^^18^ For example, a long-term study on AAV-based gene therapy in hemophilic dogs showed unique AAV integration events in the genomic DNA of treated animals, with 44% of those integrations occurring close to genes involved in cell growth.^18^ LV vectors are enveloped viruses that use single-stranded RNA (ssRNA) as the cargo with a glycoprotein envelope that facilitates attachment and entry into the host cell.^19^ Upon entering the host cell, the reverse transcriptase enzyme initiates reverse transcription, resulting in the production of double-stranded linear DNA (dsDNA). This dsDNA is then integrated into the genome by an integration enzyme and can replicate along with the genome. LV vectors can also be used in CRISPR-Cas9-based gene therapy. However, typical LVs may cause more severe off-target effects because of the incorporation of CRISPR-Cas9 into the host genome.^4^^,^^20^^,^^21^^,^^22^ The use of LNPs to deliver CRISPR-Cas9 mRNA is considered the most promising *in vivo* administration strategy. The organizational affinity can be tailored by modifying the surface of LNPs or by altering the LNP formulation.^23^^,^^24^^,^^25^ Moreover, LNPs exhibit low non-immunogenicity, are easy to assemble, form stable complexes with nucleic acids, and can be readily be scaled up for industrial commercialization.^26^

Despite the advantages of CRISPR-Cas9 mRNA therapeutics over DNA or protein-based therapies, their widespread application in biomedicine is hampered by several challenges. Firstly, mRNA has a short half-life and is susceptible to degradation due to the abundance of nucleases in the blood. Secondly, exogenous mRNA can induce a series of immune responses.^27^^,^^28^^,^^29^^,^^30^^,^^31^^,^^32^^,^^33^^,^^34^ For example, double-stranded RNA (dsRNA) activates Toll-like receptors 3 (TLR3),^27^ and ssRNA acts as a ligand for TLR7 and induces the TLR7-dependent production of inflammatory cytokines.^28^ Also, the 5′ triphosphate (5′ ppp) or diphosphate and the uridine-rich sequences can activate RIG-I.^30^^,^^31^^,^^32^ Third, unlike immunostimulant therapeutics, which only need minimal amounts of protein, gene therapies that use CRISPR-Cas9 mRNA require efficient translation in order to reach therapeutic thresholds. Therefore, improving the stability and the translation efficiency of CRISPR-Cas9 mRNA *in vivo* is necessary to further enhance its clinical outcomes.

**Figure 1:**
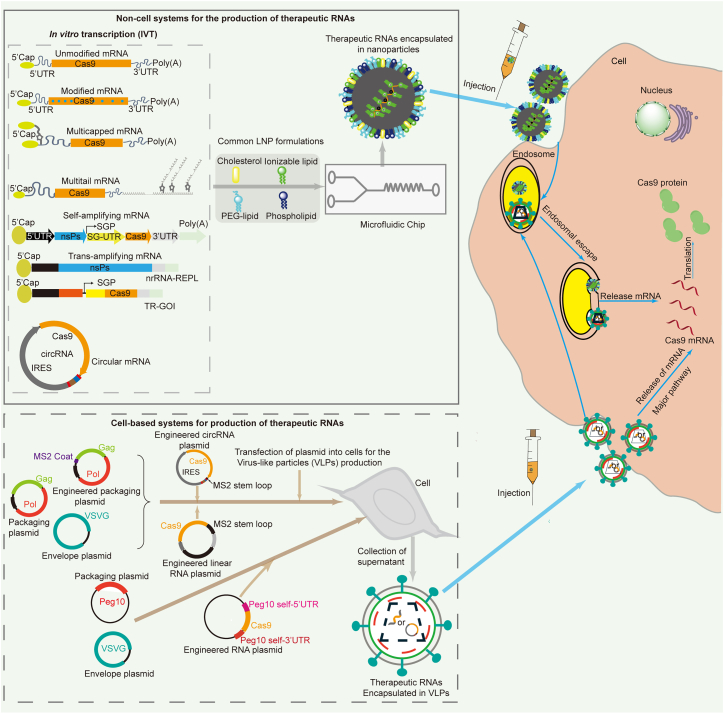
Improving mRNA-driven CRISPR-Cas9 gene therapy through mRNA engineering and the use of novel delivery vectors GOI, gene of interest; VLPs, virus-like particles; nr-RNA-REPL, non-replicative mRNA; TR-GOI, *trans*-replicon gene of interest; SEND, selective endogenous encapsulation for cellular delivery.

Several approaches can be used to enhance the therapeutic potential of CRISPR-Cas9 mRNA. These include the rational engineering of mRNA through optimizing component and chemically modifying or codon optimization, exploring novel RNA tertiary structures like circular RNA (circRNA) or self-amplifying mRNA (saRNA), and developing novel delivery systems (Figure 1). This review summarizes recent advancements in the enhancement of mRNA stability and translation efficiency as well as the development of delivery vectors for mRNA forms of CRISPR-Cas9. Moreover, the development of novel gene editors based on CRISPR-Cas9 engineering and the length fragment integration technology based on prime editing (named PE: a highly precise, search-and-replace genome editing technology) engineering and the clinical application of mRNA forms of CRISPR-Cas9 are also discussed.

## *In vivo* studies of the three cargo forms of CRISPR-Cas9-based gene therapy

The DNA forms of CRISPR-Cas9 have more stable structures and potentially longer-lasting effects compared to the mRNA and RNP forms, which often translates to more prolonged transcription and higher editing activity. AAVs, on the other hand, exhibit unique characteristics, such as minimal pathogenicity, targeted tissue tropism, and long-lasting gene expression. Thus, due to their advantageous characteristics, the combination of the DNA forms of CRISPR-Cas9 and AAVs has made them the most popular CRISPR-Cas9-based gene therapy, which has been explored for gene therapy of hearing loss,^35^^,^^36^ and in eye disease research.^5^ However, the longer-lasting transcription and translation of the DNA forms can easily trigger unexpected editing events that lead to potential risks. For example, Yeh et al., based on AAV delivery, used CRISPR-Cas9-based systems to target the *TMC1* c.A545G mutation *in vivo*, but this led to bystander edits at nearby C_10_ sites that resulted in a silent mutation.^36^ In addition, one weakness of AAV is their limited packaging capacity, which is typically around 4.7 kb, and CRISPR-Cas9-based systems may easily exceed this limit. Although dual AAV delivery strategies can overcome this packaging limitation, these come with the cost of impaired editing activity.^35^^,^^36^ Furthermore, DNA can trigger the risk of integration into the host genome,^37^ AAV can lead to integration events,^18^ and AAV-caused adverse events have been observed.^38^ For example, a high dose of rAAV9 caused capillary leakage into the lungs of the patients, resulting in lung damage and death events, thus overshadowing AAV-based gene therapy.^38^ Administering AAV-based is preferred as a local injection therapy to avoid systemic administration which may cause serious events. AAV-based gene therapy has successfully been applied in clinical studies, such as for treating hearing loss and eye-related disease.^5^^,^^39^^,^^40^

The RNP forms of CRISPR-Cas9 exhibit superior editing efficiency and the lowest off-target effects. However, the challenges with RNP forms of CRISPR-Cas9 are that they are expensive and difficult to produce, and there is currently a lack of efficient *in vivo* delivery vectors. For example, the SpCas9 protein exerts effects that require the correct three-dimensional structure, but during production, the SpCas9 protein is exposed to a series of liquid buffers. This raises concerns; how to ensure its activity is still a general challenge in the production of recombinant protein.^41^ Additionally, the lack of highly efficient delivery systems for RNP forms of CRISPR-Cas9 limits their clinical outcomes. For example, Gao et al. used the Lipofectamine 2000 to deliver RNP forms of CRISPR-Cas9 targeting the *TMC1* (p.M418K, c.T1253A) mutation as a therapy for hearing loss, but partial recovery of auditory function was only maintained for a short time (4 weeks),^9^ whereas targeting of the same locus using AAV delivery of CRISPR-Cas13 systems prevented the hearing loss for at least 12 weeks.^42^ Lipofectamine 2000 to deliver RNP forms of CRISPR-Cas9 just maintained short time; the reason is speculated to be related to low editing efficiency (<2%) caused by inefficient *in vivo* delivery. Recently, virus-like particles (VLPs) based on LV or retrovirus (Moloney murine leukemia retrovirus, MMLV) have been developed for delivering RNP forms of CRISPR-Cas9, and these have been reported to mediate efficient editing activation *in vitro* and *in vivo*.^43^^,^^44^^,^^45^^,^^46^ While VLP delivery of RNP forms of CRISPR-Cas9 holds great clinical potential, there are still safety concerns that cannot be ignored. For example, Ling et al. used VLPs to deliver CRISPR-Cas9 RNP for the treatment of the ocular neovascular damage, but this led to changes in the immune microenvironment in the eyes and induced the infiltration of a variety of immune cells.^46^

The mRNA forms of CRISPR-Cas9-based gene therapy combine the advantages of the previously described forms while circumventing the limitations of the DNA and RNP forms of CRISPR-Cas9. For example, RNA eliminates the risk of the host genome integration, which is present in DNA forms. mRNA, through direct translation in the host cell, solves the obstacles of post-translational modification, folding, assembly, and location of exogenously expressed proteins.^47^ Meanwhile, mRNA can encode multiple proteins composed of multiple subunits, which solves correct three-dimensional structure of exogenously expressed proteins. Furthermore, the production and manufacture of mRNA are faster, more convenient, and less expensive than of protein drug.^47^ Although protein-based therapeutics have existed for a long time, with more mature regulatory frameworks and approval experience compared to mRNA drugs, RNA drugs have garnered significant attention in recent years due to breakthrough advances in LNP technology. The natural tendency of LNPs to accumulate in the liver has sparked the interest of current clinical studies for LNP-mediated delivery of mRNA forms of CRISPR-Cas9 mainly for liver-related diseases, such as the NTLA-2001 trial^48^ and the VERVE-101 trial.^8^^,^^49^ Therefore, currently, we consider that the optimal solution for CRISPR-Cas9-based gene therapeutic should be based on LNP delivery of mRNA forms, particularly for treating liver-related disease.

## Improving mRNA stability and translation efficiency by engineering the mRNA components

**Figure 2:**
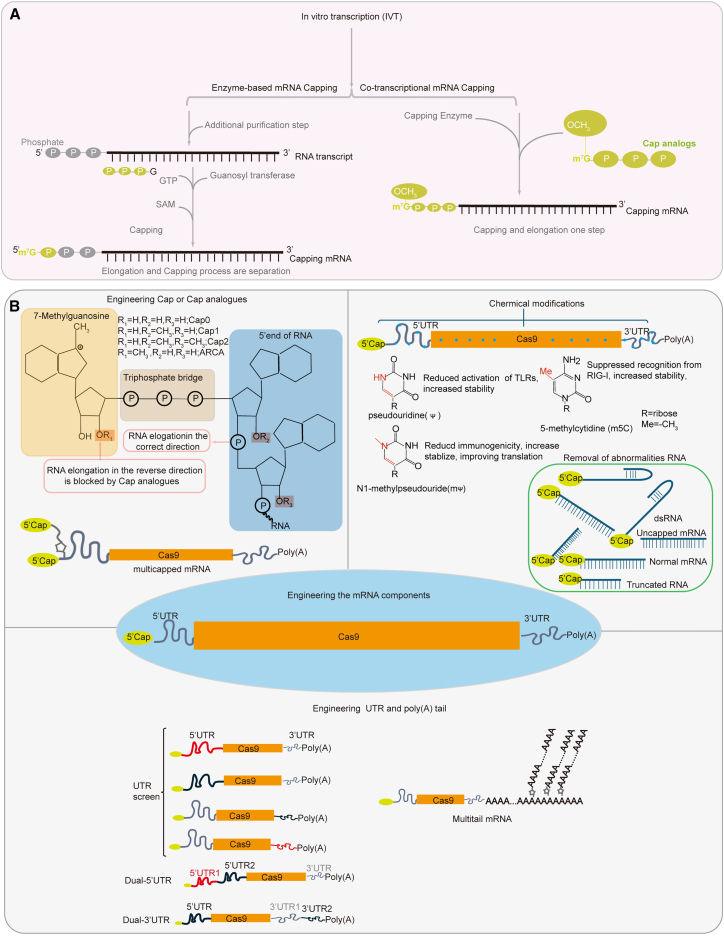
Engineering the mRNA components for improving translation capacity (A) Enzyme-based capping (left) process, which is performed after *in vitro* transcription using 5′-triphosphate RNA, GTP, and SAM, while co-transcriptional capping (right) uses an mRNA cap analog without additional purification steps. (B) Engineering of mRNA components for improving stability and translation efficiency, including engineering the cap, UTR, and poly(A) tail, incorporating modified ribonucleotides into RNA and removal of abnormalities in RNA. ARCA, anti-reverse cap analog.

Structural elements play important roles in the regulation of mRNA translation and stability.^50^ To enhance mRNA stability and translation, researchers have used rational design strategies and have focused on mRNA structural elements, including engineering of the 5′ cap, the 5′ UTR (untranslated region), the open reading frame (ORF), the 3′ UTR, and the poly(A) tail (Figure 2).

## Rational engineering of the 5′ cap to improve mRNA translation

The 5′ cap is intimately involved in RNA processing, including nuclear export and the initiation of translation, while also protecting RNA from recognition by the innate immune system and preventing mRNA degradation by 5′-3′ exonucleases.^51^^,^^52^^,^^53^ In capped mRNAs, the most common cap structure is N^7^-methylguanosine (m^7^G) linked by a 5′ to 5′ ppp bridge to the adjacent nucleotides, m^7^G(5′)p_3_N (Cap0). The adjacent nucleotides can be further methylated at the ribose O-2′ position of the first and second nucleotides, and these modifications are named m^7^G (5′) p_3_Nm (Cap1) and m^7^G (5′) p_3_NmNm (Cap2), respectively.^54^^,^^55^^,^^56^ Two typical capping methods are commonly used in *in vitro* transcription (IVT) including enzymatic capping and co-transcriptional capping (Figure 2A). Enzymatic capping process involves two enzymes and a series of reactions.^57^^,^^58^^,^^59^ Moreover, enzymatic capping methods require additional purification steps to remove residual DNA templates, dNTPs, and enzymes form IVT reaction (Figure 2A). Enzymatic capping uses an RNA triphosphatase, guanosine triphosphate (GTP), 2′-0-methyltransferase, and S-adenosylmethionine (SAM) and occurs through four processes: (1) RNA triphosphatase removes the γ-phosphate from the 5′ ppp RNA; (2) GTP couples to the removed γ-phosphate of the 5′ ppp RNA; (3) a methyl group from SAM transfers to the terminal guanine to generate Cap0; and (4) 2′-0-methyltransferase methylates the 2′ OH of the penultimate guanine to produce Cap1. Enzymatic capping allows for the easy and complete capping of the 5′ end of mRNAs. However, this method requires an additional purification step, which increases the complexity of the preparation and higher cost. Co-transcriptional capping RNA synthesis is achieved when RNA polymerase initiates transcription via nucleophilic attack of the 3′ OH of the guanosine cap analogs on the α-phosphate of the next nucleoside triphosphate specified by the DNA template.^60^^,^^61^^,^^62^^,^^63^^,^^64^^,^^65^^,^^66^ Co-transcriptional capping RNA synthesis is one step without additional purification steps, thus simplifying the experimental procedure (Figure 2A). However, two challenges with co-transcriptional capping are that competitive addition of GTP and cap analogs to the 5′ end of mRNA during transcription can occur; thus, some uncapped mRNA can also be generated, and nucleophilic attack can also occur by the m^7^G, producing transcripts capped in a reverse orientation (Figure 2B). Uncapped mRNA and reverse-capped mRNA decrease the overall translational ability of mRNA. Anyway, those problems are easily overcome; for example, anti-reverse cap analog (e.g., 7-methyl [3′-O-methyl] GpppG and 7-methyl [3′-deoxy] GpppG)-capped luciferase mRNAs show more than a 2-fold increase in translation efficiency compared to the conventional cap.^67^ It has been reported that the recently developed CleanCapAG Cap analog (m^7^G(5′)ppp(5′) (2′OMeA)pG) results in 94% or higher Cap1 mRNA during synthesis.^60^

Studies have shown that mRNA translation levels can be refined through rational engineering of the 5′ cap.^68^^,^^69^^,^^70^^,^^71^ For example, Grzela et al. developed N2-modified dinucleotide cap analogs and tested translation levels in 293HEK cells. This analog increased cap incorporation in the correct orientation and facilitated more efficient translation than the conventional cap structure.^69^ Chen et al. used a ligation-enabled mRNA-oligonucleotide assembly strategy to develop multi-capped mRNA that showed greater translation ability compared to the traditional single-cap structure of mRNA and enhanced protein production by up to 10-fold *in vivo* (Figure 2B).^68^ Klocker et al. reported on 5′ cap analogs with photo-cleavable groups, which they referred to as FlashCaps (DMNB-Cap or NPM-Cap).^71^ The FlashCap-mRNA efficiently impedes the interaction with cap-binding proteins and cap-degrading enzymes, and this silences translation in cells. Irradiation of FlashCap-mRNA by light releases Cap0-mRNA, which triggers translation in cells. Therefore, the application of FlashCap to prepare Cas9 mRNA might facilitate tissue-specific editing by leveraging spatiotemporal regulation of mRNA activity through irradiation. These results imply that engineering the 5′ cap can not only increase the translation efficiency of mRNA, but it can also mediate tissue-specific expression.

## Optimizing the 5′ UTR to improve mRNA translation

The 5′ UTR affects ribosome binding to mRNA in the proximity of the initiation codon and regulates translation through factors such as length, secondary structure, upstream AUG codons (uAUGs), and upstream ORFs (uORFs).^50^^,^^72^^,^^73^^,^^74^^,^^75^ The 5′ UTR in human mRNA typically ranges between 100 and 210 base pairs (bp),^50^ and the length of the 5′ UTR influences translation by affecting the energy required for the ribosome to navigate through the highly structured 5′ UTR in order to reach the AUG initiation codon.^50^ The stem-loop structure present in the 5′ UTR can stall the scanning by the 40S ribosomal subunit, and this increases its “dwell time” and thus reduces translation efficiency.^73^ Approximately 50% of the 5′ UTRs in mammalians mRNAs contain uORFs and/or uAUGs, both of which can modulate initiation from the main ORF, thereby influencing translation efficiency.^72^ Translation in eukaryotes is initiated at the first AUG codon, and the sequences flanking this AUG codon are non-random. This specific sequence context is known as the Kozak sequence (gccRccAUGg, R:A/G).^76^ It is thought that these flanking sequences modulate the extent to which AUG affects the translation rate.^76^ Therefore, in the design and selection of 5′ UTRs for CRISPR-Cas9 mRNA, it is advisable to avoid excessive lengths and the presence of uORFs and uAUGs. Instead, the introduction of a Kozak sequence flanking the AUG codon is recommended. Currently, the commonly utilized 5′ UTR is human alpha globin, which is 37 nucleotides in length.^77^ Many studies have sought to identify 5′ UTRs that are capable of more efficiently regulating mRNA translation. For example, Trepotec et al. developed a minimalistic 5′ UTR consisting of 14 nucleotides by combining the T7 promoter with a Kozak consensus sequence.^78^ The 5′ UTR mediated similar or even higher expression levels than the human alpha globin 5′ UTR. Asrani et al. performed an *in vitro* screening based on a library of ten 5′ UTR variants and found two 5′ UTR variants that showed a higher translation efficiency translation than human alpha globin 5′ UTR.^79^ Le et al. constructed dual 5′ UTRs in bacterial expression systems (Figure 2B). These dual 5′ UTRs showed synergistic effects on the protein expression and exceeded the protein levels achieved with a single 5′ UTR.^80^ Sultana et al. on the other hand, identified a highly efficient 5′ UTR from the fatty acid metabolism gene carboxylesterase 1D (Ces1d), which increased expression levels about 2-fold in the heart post myocardial infarction compared to an artificial 36-nucleotide 5′ UTR.^81^ Further analysis revealed that the Ces1d 5′ UTR also enhanced translation in the liver but not in the kidney. It is suggested that optimizing the 5′ UTR can improve mRNA translation efficiency and regulate tissue-specific expression as well.

## Engineering the 3′ UTR and poly(A) tail to improve mRNA translation

The 3′ UTR is situated downstream of the terminal codon and plays an important role in the regulation of various mRNA processes, including translation, stability, and localization.^82^^,^^83^^,^^84^^,^^85^ The translation rate of mRNA is influenced by the length of the 3′ UTR and by the presence of negative regulatory elements. For instance, mRNAs with longer 3′ UTRs have shorter half-lives, while those with shorter 3′ UTRs are inefficiently translated.^86^^,^^87^ AU-enriched elements, GU-enriched elements, and microRNA-binding sites in the 3′ UTR contribute to reduced mRNA stability.^88^^,^^89^^,^^90^ Currently, the 3′ UTR sequences that are widely used in *in vitro* preparations of mRNAs are derived from α-globin and β-globin and incorporate regulatory elements that regulate translation and stability.^91^^,^^92^ Numerous studies have sought to identify 3′ UTRs that are capable of efficiently mediating mRNA translation. For instance, Holtkamp et al. showed that sequentially incorporating two β-globin 3′ UTRs enhances mRNA stability and translational efficiency than without any 3′ UTR or with only a single β-globin 3′ UTR (Figure 2B).^93^ Alexandra et al. applied a cell-based selection process to identify two 3′ UTRs, namely mtRNR1 and AES, which significantly improved expression levels compared to β-globin 3′ UTR in mRNA.^94^

The poly(A) tail at the 3′ terminus of mRNAs is essential for mRNA transport, translation, and stability.^95^^,^^96^ The broadly accepted mechanism of cap-dependent translation is that the poly(A) tail indirectly binds to the 5′ cap via eukaryotic translation initiation factors and poly(A)-binding protein, resulting in the formation of a multimeric complex and a closed-loop structure.^97^^,^^98^^,^^99^ Additionally, the binding of poly(A)-binding protein to poly(A) tails requires a minimum of 27 adenines.^100^ Therefore, mRNAs with short poly(A) tails (<50 nucleotides) are typically translationally repressed, and removal of the poly(A) tails leads to decapping (cap removal) and exonuclease-mediated degradation.^101^ Protecting poly(A) tails from degradation can enhance mRNA translation, for example, ligation of oligonucleotides to the poly(A) terminus increases protein production by 2- to 10-fold in HeLa cells and neuronal cultures.^102^ Chen et al. reported on ligation of modified branched poly(A) oligonucleotides to generate multi-tail mRNA, which enhanced protection against RNA decay and prolonged translation for up to 14 days (Figure 2B).^103^ A longer poly(A) tail is not necessarily better. Typically, an mRNA poly(A) sequence of 120 units provides sufficient stability and efficient translation. However, whether the poly(A) tail should be shorter or longer seems to be transcriptionally specific.^93^^,^^104^^,^^105^

## Engineering the ORF to improve mRNA translation

Exogenous mRNA is inherently recognized by innate immune receptors and stimulates the immune system.^27^^,^^28^^,^^29^^,^^30^^,^^31^^,^^32^^,^^33^^,^^34^ In the past decade, studies have shown that chemical modification, codon optimization, and the removal of dsRNA from mRNA samples can reduce the activation of the innate immune system, enhance both mRNA stability, and improve translational efficiency. The preparation of mRNA using modified nucleosides to attenuate the immunostimulatory response and improve mRNA stability and translation levels has been widely discussed.^33^^,^^106^^,^^107^^,^^108^^,^^109^^,^^110^^,^^111^^,^^112^ For instance, Karikó et al. investigated how naturally modified RNA influences immunostimulatory effects.^112^ They found that RNA containing m6A, 5-methylcytidine, 5-m5U, pseudouridine, or 2-thiouridine modifications can limit or abolish the activation of TLRs. Kormann et al. found that replacing 25% of the uridines and cytidines with 2-thiouridine and 5-methylcytidine synergistically decreased mRNA binding to TLR3, 7, and 8 and RIG-1 receptors.^107^ Jiang et al. reported an approximately 1.5-fold greater editing efficiency *in vivo* compared with unmodified adenine base editor (ABEs) by using 5-methoxyuridine modifications of the ABE for editing of the *Fah* gene.^113^ Haideri et al. used N1-methyl-pseudo-UTP modifications of ABE8e targeting the *B2M* gene in human pluripotent stem cells and achieved a significantly greater knockout efficiency compared with plasmid-based systems.^114^ Vaidyanathan et al. replaced synonymous codons to generate a uridine-depleted Cas9 ORF, which showed lower immune responses compared with the wild-type Cas9.^106^ They also explored modified nucleosides, codon optimization, and the removal of structural abnormalities in RNA. Codon optimization and 5-methoxyuridine and N1-methyl-pseudo-UTP chemical modifications of Cas9 mRNA resulted in greater editing activity compared to wild-type Cas9 mRNA. The removal of Cas9 double-stranded RNA resulted in reduced immune responses and increased editing efficiency compared to untreated wild-type Cas9 mRNA. The predominant modified nucleosides currently incorporated into therapeutic and vaccine mRNA sequences include pseudouridine, N1-methylpseudouridine (m1Ψ), and 5-methylcytidine^115^^,^^116^ (Figure 2B).

## Rational design through structural engineering to enhance mRNA translation

**Figure 3:**
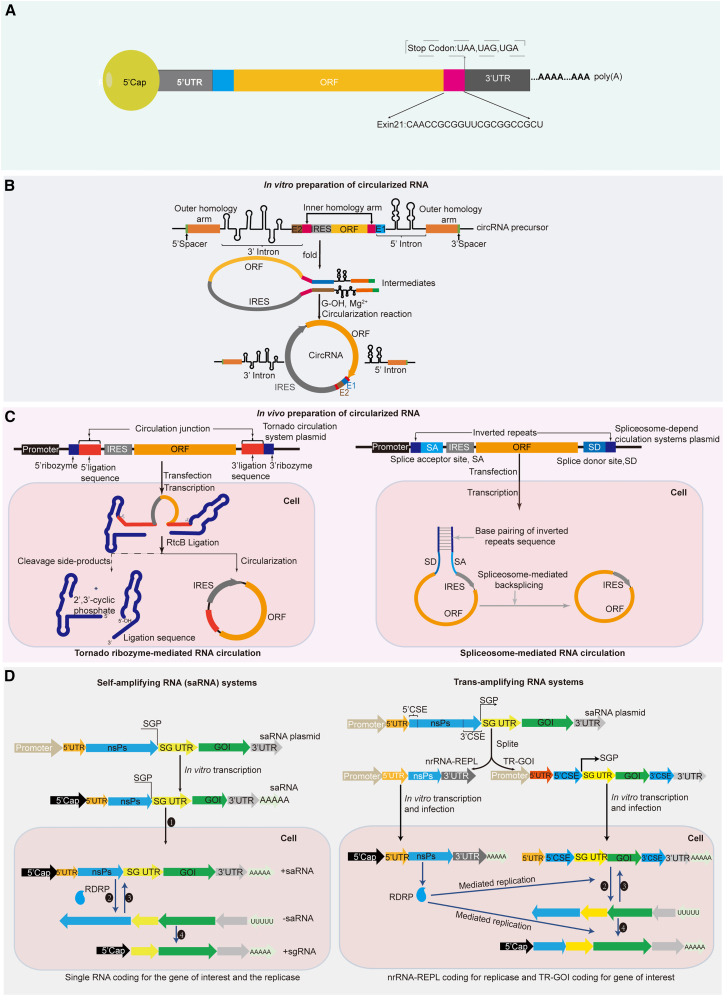
Structural engineering of mRNA to enhance translation efficiency (A) Exin21 fusion to the ORF mediates mRNA translation and stability. (B) Schematic of the *in vitro* preparation of circRNA. The diagram shows elements of engineering the self-splicing precursor RNA and the circularization process. (C) Schematic of the in *vivo* preparation of circRNA. On the left is the Tornado system for the preparation of circRNA in the cell. On the right is spliceosome-dependent preparation of circRNA *in vivo*. (D) Schematic of saRNA self-replication *in vivo*. On the left is the single saRNA system. (1) saRNA enters the cell translation RDRP; (2) RDRP amplifies the +saRNA to produce −saRNA; (3) RDRP amplifies the −saRNA to produce +saRNA; (4) RDRP amplifies the −saRNA at the SGP site to produce to +sgRNA. On the right is the *trans*-amplifying RNA systems. *Trans*-amplifying splits nrRNA-REPL and TR-GOI from saRNA plasmid. The nrRNA-REPL plasmid encodes a replicase, and the commonly used 5′ UTR is human alpha globin, while its 3′ UTR (3′AES/mtRNR1) is a fusion of motifs derived from amino terminal enhancer of split (AES) mRNA and mitochondria-encoded 12S rRNA (mtRNR1). TR-GOI plasmid coded for the gene of interest, and its UTR is from the saRNA itself; CSEs are the conserved sequence elements, 5′CSE:221nt and 3′CSE 2952 nt and these are necessary to ensure TR-GOI RNA replication by the replicase.

In addition to engineering the mRNA components to improve translation, there have been studies on enhancing mRNA stability and translation efficiency through structural engineering. For example, Zhu et al. found that insertion of a 21-mer *cis*-regulatory motif (Exin21) in the ORF significantly enhanced the expression of the gene of interest (GOI)^117^ (Figure 3A). Further validation by mRNA decay assays suggested that Exin21 enhances mRNA translation by increasing the half-life of nascent mRNA by approximately 3- to 6-fold.

Generally, traditional linear mRNA molecules are easily degraded by RNases through either 5′-3′ RNA decay or 3′-5′ RNA decay.^95^ Translatable circRNA are ssRNA molecules that are covalently linked in a head-to-tail manner, thus avoiding exposure of the 5′ and 3′ ends. As a result, circRNA has increased stability, a longer half-life, and a higher level of translation abundance compared to linear RNA.^118^^,^^119^^,^^120^ The permuted intron-exon (PIE) system is widely used to produce exogenous circRNA. The PIE circularization strategy only requires the addition of GTP and Mg^2+^ as cofactors to mediate the autocatalytic ribozymatic reactions that ligate flanking segment sequences during the generation of circRNA^121^^,^^122^ (Figure 3B). The circRNA can utilize a cap-independent translation mechanism by incorporating an internal ribosome entry site (IRES) upstream of the GOI, which consequently recruit ribosomes to initiate translation.^121^ Li et al. compared the translation differences between RNA^D2GFP^, M1ΨmRNA^D2GFP^ (M1Ψ:N1-methylpseudouridine), and circRNA^D2GFP^ and found that circRNA^D2GFP^ had greater stability and greater expression compared with other two groups.^118^ Chen et al. optimized the essential elements in order to maximize the circRNA translation rate, and this resulted in several 100-fold improvements in circRNA protein yields.^119^ However, the insertion of a minimal-size exogenous sequence (i.e., E1, E2, and spacer) in the circRNA is unavoidable when using the PIE circulation strategy (Figure 3B). Liu et al. showed that the exogenous sequence (i.e., E1, E2, and spacer) can distort the folding status of the original circRNA or can itself form structures that provoke a more innate immune response compared to the synthesis of circRNA by T4 RNA ligase (T4 RNA ligase without extraneous fragments produces).^123^ To avoid insertion of an exogenous sequence, Qiu et al. have described a novel circulation strategy, namely Clean-PIE.^120^ The Clean-PIE circularization strategy is built upon the PIE strategy and induces the concealing of the E1 and E2 sequences within the ORF or IRES sequences, thus eliminating the need to produce exogenous sequences. The Clean-PIE approach can achieve a circularization rate of approximately 90% for short RNAs of around 2,000 bp in size and has been shown to result in sustained protein expression and reduced immunogenicity both *in vitro* and *in vivo*.

However, *in vitro* transcription preparation of circRNA requires multiple steps and a nuclease-free environment. Unti et al. achieved in-cell synthesis of circRNA based on the adaptation of the Tornado (Twister-optimized RNA for durable overexpression) system (Figure 3C).^124^ The Tornado system involves the synthesis of a linear RNA containing ribozymes at the 5′ end and 3′ end of the transcript. After the ribozymes undergo autocatalytic cleavage, the 5′ and 3′ ends are ligated by the ubiquitous endogenous RNA ligase RtcB.^124^^,^^125^ This ligation results in the production of circRNA in the cell. Experimental results indicate that the Tornado system can circularize mRNAs as large as approximately 5,000 bp. Although the Tornado system produces circRNA with lower protein abundance than linear mRNAs, it maintains more sustained levels of protein production compared to linear mRNA translation systems. Wang et al. reported a spliceosome-dependent production method of circRNAs *in vivo*^126^ (Figure 3C). Splicesome-dependent RNA process is by joining a upstream 3′ splice sites (SA: splice acceptors) and a downstream 5′ splice sites (SD: splice donor sites) in GOI, process called backsplicing. Pasman and Dubin et al. further found that inverted repeats flanking the splice acceptor and splice donor sites are necessary for circularization; thus, the design of the spliceosome-dependent circularization systems commonly adds inverted repeats sequences.^127^^,^^128^

Although increasing the amount of mRNA administered in a single dose can increase protein expression, this approach faces inherent limitations, including constraints imposed by the minimum safe dose threshold and the linear relation between mRNA quantity and antigen expression. saRNA overcomes this limitation by requiring only a minimal dosage for efficient expression, while reducing side effects and achieving long-lasting outcomes.^129^^,^^130^^,^^131^^,^^132^^,^^133^ saRNA is derived from the bicistronic genome of plus-stranded RNA viruses, such as alphaviruses and flaviviruses.^134^^,^^135^^,^^136^ The most commonly used viruses in saRNA research are Venezuelan equine encephalitis virus (VEEV), Semliki Forest virus (SFV), and Sindbis virus.^136^^,^^137^^,^^138^ saRNA shares many structural similarities with linear mRNA, and it is an ssRNA molecule consisting of a 5′ cap, a 5′ UTR, gene coding for non-structural proteins (nsPs), an SGP (subgenomic promoter), an SG-UTR (subgenomic UTR), a GOI, a 3′ UTR, and a poly(A) tail (Figure 3D). The nsPs consist of four subunits: nsP1, nsP2, nsP3, and nsP4, which encode the replicase complex, i.e., the RNA-dependent RNA polymerase (RDRP). Upon entering the cytoplasm, saRNA is translated to produce RDRP. RDRP amplifies the original saRNA strand (i.e., +saRNA) to produce the complementary negative saRNA (−saRNA) strand. Simultaneously, RDRP is capable of amplifying −saRNA to produce +saRNA. Additionally, RDRP recognizes the SGP sequence, which mediates the downstream sequence amplification to produce +sgRNA (Figure 3D).

While the advantages of saRNA are significant compared to linear mRNA, the insertion of exogenous sequences exceeding 2,000 bp results in an saRNA length that exceeds 10,000 bp.^139^^,^^140^ The excessive length and complexity of mRNA sequences make them susceptible to degradation by nucleases, and these also pose challenges for mRNA preparation and delivery.^130^^,^^141^ Beissert et al. developed a novel saRNA system (referred to as the *trans*-amplifying RNA system, taRNA) that not only overcomes the limitations of excessive length and complexity but also mediates more efficient translation compared to typical saRNA.^142^ taRNA is a split vector system consisting of both *trans*-replicon-GOI (TR-GOI) and non-replicative mRNA (referred to nrRNA-REPL) vectors (Figure 3D). The TR-GOI encoding the GOI is engineered from saRNA by incompletely deleting nsPs while retaining the 5′ conserved sequence element (5′ CSE), as well as the SGP, SG-UTR, and 3′ CSE of the SFV. The 5′ CSE consists of 221 bp of nsP1 and the 3′ terminal 984 bp of nsP4. The replicase activity is encoded on the nrRNA-REPL RNA molecule. The nrRNA-REPL structure uses typical RNA elements, such as the conventional 5′ cap, and 5′ UTR of human alpha-globin. VEEV-based taRNA systems have also been tested. Unlike the SFV, the TR-GOI vector of VEEV only contains the 5′ terminal 141 nucleotides of nsP1 as its 5′ CSE.^134^ However, the innate immune response of host cells and the inherent instability of RNA inevitably lead to a gradual decline in RDRP. Li et al. attempted to address these shortcomings, screening two mutations at nsP2 and nsP3 that showed prolonged duration and expression of saRNA *in vivo*.^143^

## Delivery vectors for mRNA

**Figure 4:**
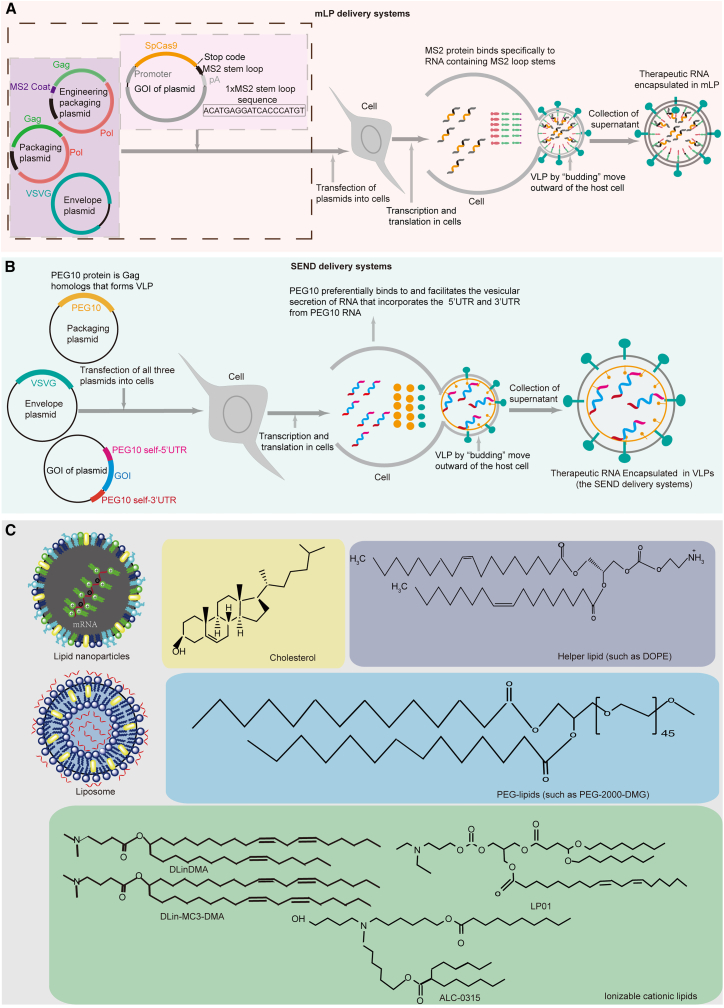
mRNA delivery platforms (A) Schematic of the mLP delivery platform for mRNA. The engineering packaging plasmid was deisgned by inserted the MS2 protein at the N terminus of Gag-pol, and Gag-pol is the packaging plasmid. The target plasmid is inserted with an MS2-stem-loop between the stop codon and the poly(A) signal sequence(pA). The mLP delivery system is based on LV by engineering a packaging plasmid that can encapsulate the mRNA of interest to produce VLPs. (B) Schematic of the SEND delivery platform for mRNA. The PEG10 protein is a Gag protein homolog that can specifically identify and bind to self-untranslated regions. (C) The structure of LNPs and liposomes. LNPs have a phospholipid monolayer structure that usually contains cationic lipids and helper lipids, cholesterol, and PEGylated phospholipids. The liposomes have a phospholipid bilayer structure that usually contains phospholipids and cholesterol and may also contain other polymers and even membrane proteins.

Delivery vectors play an important role in the success of mRNA-driven CRISPR-Cas9 gene therapy, and the lack of safe and efficient delivery vectors has become a critical challenge in the clinical application of such systems. Currently, common delivery vectors for mRNA include those based on LV or retroviral vectors rationally redesigned from VLPs, extracellular vesicles (EVs), LNPs, liposomes nanoparticles, polymer nanoparticles, etc (Figure 4).

## VLPs as delivery vectors

LV delivery vectors are not the primary platform for CRISPR-Cas9 delivery due to their potential to facilitate the integration of exogenous sequences into the host genome. However, LVs can be modified through strategic engineering to render them incapable of genome integration while retaining their capacity to bind to specific mRNAs and to maintain infectivity.^144^ Ling et al. engineered LVs by fusing a monomer MS2 coat (MS2C) protein (bacteriophage-derived MS2 protein) to the N terminus of the Gag-pol. While working with the mRNA of interest, they inserted a six-repeat stem structure between the stop codon and the poly(A) tail. The MS2C protein can specifically recognize this stem structure (named MS2 stem loop), thus enabling specific mRNA encapsulation. The novel delivery system was termed mLP^144^ (Figure 4A). Subsequently, based on the mLP platform, CRISPR-Cas9 mRNA (mLP-CRISPR) was delivered to target vascular endothelial growth factor A (*Vegfa*) for the treatment of wet age-related macular degeneration in a mouse model. A single subretinal injection of mLP-CRISPR knocked out 44% of Vegfa in the retinal pigment epithelium, without inducing off-target editing or anti-Cas9 immune responses. Ling et al. have also used the mLP-CRISPR-based delivery platform for targeting the *UL8* and *UL29* genes in the *HSV-1* genome for treatment of herpetic stromal keratitis in mice.^145^ A single corneal injection of mLP-CRISPR induced an editing efficiency of about 7% for the *UL8* gene and 5% for the *UL29* gene, and no off-targets effects were seen for either sgRNA. Unti et al. similarly used the mLP-based delivery platform to perform intracellular synthesis and packaging of circRNAs.^124^ Segel et al. found that Gag homolog-retroviral-like proteins (PEG10) can specifically bind to self-untranslated regions.^146^ Based on this property, they developed the selective endogenous encapsulation for cellular delivery (SEND) systems, which flanks the mRNA of interest with the UTR of PEG10 (Figure 4B). Using the SEND delivery system for CRISPR-Cas9 mRNA showed that it mediates robust editing activity.

## EVs as delivery vectors

EVs have recently emerged as a viable platform for mRNA delivery.^147^^,^^148^^,^^149^ EVs are endogenously produced by cell and thus exhibiting lower inflammatory responses, and they inherit the properties of the parental cells. Therefore, EVs are ideal candidates for engineering targeted and specific delivery vectors that allow for repeated dosing for therapeutic applications.^146^^,^^148^^,^^150^^,^^151^^,^^152^ Despite the significant advantages of EVs in terms of biocompatibility and hypo-immunogenicity, a major drawback that urgently needs to be addressed is to remove the encapsulation of large amounts of heterogeneous mRNA within EVs.^147^

## LNPs as delivery vectors

LNPs are among the most promising RNA delivery platforms and have been approved by the FDA for mRNA drug delivery.^153^^,^^154^^,^^155^^,^^156^^,^^157^^,^^158^ LNPs are biocompatible vehicles with a phospholipid monolayer structure that can electrostatically encapsulate mRNAs within the LNPs, thus protecting the mRNA from degradation by RNases (Figure 4C).^159^^,^^160^ LNP formulations usually contain cationic lipids or ionizable cationic lipids and helper lipids, cholesterol, and PEGylated phospholipids.^161^^,^^162^^,^^163^^,^^164^ Among these, the most critical components are the cationic lipids or ionizable cationic lipids, which are decisive factors for encapsulating mRNA in the LNPs and for facilitating the escape of nanoparticles from endosomes, both of which affect the delivery and transfection efficiency of mRNA.^165^^,^^166^ The helper lipids strengthen the overall phase transition temperature and stability of the LNPs and aid in endosomal escape.^161^^,^^162^^,^^163^^,^^164^ Cholesterol contributes to the stability of the lipid layer of LNPs and enhances the intracellular intake and cytoplasmic entry of mRNA.^161^^,^^162^^,^^163^^,^^164^ PEGylated phospholipids located on the surface of the LNPs improve the hydrophilicity of LNPs, reduce LNP aggregation, prevent clearance by the immune system, and increase the stability of LNPs.^161^^,^^162^^,^^163^^,^^164^

In the early research, cationic lipids were commonly used in LNP formulations to deliver mRNA. For example, Rejman et al. utilized 1,2-dioleoyl-3-trimethylammonium-propane (DOTAP) as a cationic lipid in LNPs to deliver mRNA to carcinoma cell and mesenchymal stem cells.^167^ Lou et al. conducted a comparative analysis of the delivery effects of commonly available cationic lipids for saRNA delivery.^168^ The cationic lipids they evaluated included 3β-[N-(N′,N′-dimethylaminothane)-carbamoyl]cholesterol, dimethyldioctadecylammonium (DDA), 1,2-stearoyl-3-trimethylammonium-propane, 1,2-dimyristoyl-3-trimethylammonium-propane, N-(4-carboxybenzyl)-N, N-dimethyl-2,3-bis (oleoyloxy) propane-1-aminium, and DOTAP. Among these, the helper lipid 1,2-dioleoyl-sn-3-phosphoethanolamine in combination with DOTAP or DDA resulted in the most efficient delivery. Kedmi et al. investigated the potential risks of cationic lipids containing LNPs *in vivo*.^169^ Through the comparative analysis of neutral or negatively charged lipids, they showed that cationic lipids resulted in increased hepatotoxicity and provoked immunological responses, and this was attributed to their strong positive charge and their inability to escape from endosomes.^169^^,^^170^ In order to bypass these challenges, ionizable cationic lipids have been widely investigated for mRNA delivery.^153^^,^^154^^,^^171^ These ionizable cationic lipids have the ability to alter their charge in response to pH changes, thus offering advantages in mRNA delivery by minimizing toxicity and enhancing endosomal escape through mechanisms such as the “flip-flop” or “proton sponge” effect.^165^ The first FDA-approved LNP delivery system using ionizable lipids (e.g., 1,2-dilinoleyl-*N*,*N*-dimethyl-3-aminopropane) was designed to use RNAi to treat transthyretin-mediated amyloidosis.^157^ Since then, LNP technology has sparked significant interest as a delivery platform for gene editing tools and mRNA vaccines. For example, Finn et al. developed LNP vectors containing the ionizable LP01 lipid for delivering CRISPR-Cas9 mRNA targeting the *TTR* gene to treat a mouse model of transthyretin amyloidosis.^7^ In that study, a single administration induced a 97% reduction in TTR protein, and the effect persisted for at least 1 year. Rothgangl et al. developed LNPs using the ionizable cations for delivering the base editing tools (ABEs) targeting *PCSK9* in order to reduce low-density lipoprotein (LDL) levels. Their LNPs resulted in 67% editing in mice and up to 34% editing in macaques.^8^^,^^172^^,^^173^ The Moderna and BioNTech/Pfizer COVID-19 vaccines mRNA-1273 and BNT162B2 also utilized ionizable cationic lipids (e.g., Dlin-MC3-DMA and ALC-0135, respectively) in their LNP formulations.^159^

## Liposomes as delivery vectors

Liposomes have also been extensively studied as nanoparticle-based vectors for nucleotide delivery. Distinct from the monolayer structure of LNPs, liposomes have a phospholipid bilayer structure^159^^,^^174^ (Figure 4C). The double-layered lipid membrane structure of liposomes closely resembles the cell membrane, making it easy for liposomes to fuse with cells and thus achieve high transfection efficiency. In addition, liposomes can leverage various stimuli like light, pH, ultrasound, magnetism, etc., to spatiotemporal control of CRISPR-Cas9-based drug releases, thus increasing editing efficiency and reducing off-target effects.^174^^,^^175^ For example, Aksoy et al. developed light-triggered liposomes by incorporating a photosensitive molecule into the lipid bilayer delivery of RNP forms of CRISPR-Cas9. Under light illumination at 690 nm, the liposomal structure is destabilized resulting in CRISPR-Cas9 release.^176^

## Polymer nanoparticles as delivery vectors

Polymer nanoparticles represent another class of mRNA delivery vehicles. Polymeric nanoparticles containing flexible and long polymer chains with diverse chemical structures can engage in electrostatic attraction, hydrophobic interactions, and additional physical entanglements, consequently making them more stable than many other nanoparticles.^177^^,^^178^^,^^179^ For example, the polyethyleneimine (PEI) polymer family consists of a variety of linear and branched members. Branched PEIs have strong ionic charges, and this facilitates efficient packaging by compacting negatively charged nucleic acids into multiplexes that shield nucleic acids from degradation by nucleases, and the pH-buffering ability of these vectors aids in the intracellular endosomal escape of nucleic acids.^180^ Cheng et al. used an anionic di-block co-polymer (PEG-PLE) and lipid-modified PEI (C14-PEI) nanoplexes to deliver Cas9 mRNAs and sgRNA-targeting KRAS^G12S^ for lung cancer therapy.^181^

## Peptide-based delivery vectors

In recent years, peptide-based RNA drug delivery platforms have shown great potential. Therapeutic peptides are usually 7–50 amino acids in length and exhibit a cationic charge state. Peptide-based RNA drug delivery platforms are divided into stand-alone carrier and those combined with other carriers. In stand-alone carrier platforms, the positively charged cationic peptides condense the negatively charged mRNAs into nanoparticle size through electrostatic interactions.^182^^,^^183^ For example, Gonzalez et al. simply mixed ADGN peptides and RNA forms of CRISPR-Cas9 to form stable nanoparticles.^184^ An effective targeted gene editing *in vivo* lung cells was achieved after systemic intravenous administration of the ADGN-CRISPR-Cas9 complexes. Foss et al. used A5K peptides by simply mixing with a CRISPR-Cas9 RNP to developed peptide-based delivery platform, which mediated highly efficient editing in T cell.^185^ Peptides with tissue-specific targeting capabilities are often used to modify the surface of delivery vectors in order to further enhance delivery performance or tissue specificity. For example, LNP delivery of mRNA is restricted to the retinal pigment epithelium and the Müller glia and cannot transfect visual phototransduction cells.^186^ Barrera et al. used phage display technology to screen for peptides, and LNP decorated with candidate peptide ligands for delivery of mRNA showed robust protein expression in visual phototransduction cells.^186^

## Target-specific mRNA delivery

Efficient and targeted delivery to specific cells and tissues determines the therapeutic efficacy and minimizes the off-target side effects of mRNA drugs.^187^^,^^188^ The most popular LNP-based mRNA delivery vectors have a tendency to accumulate in the liver, and bypassing the liver to targeting various organ is necessary to maximize the potential of LNP-based mRNA therapeutics. Numerous efforts to bypass the liver have been made in LNP-based mRNA delivery platforms. Cheng et al. added a fifth charged molecule to the traditional four LNP molecules, and this achieved specific mRNA delivery to the lung and spleen, where the fifth molecule was a cationic lipid or an anionic lipid, respectively.^23^ Dietmarir et al. reported a cell-specific mRNA delivery LNP using bi-specific antibodies that bound to PEG on the exterior of the LNP.^187^ By modifying the epidermal growth factor receptor and folate hydrolase 1 (PSMA) of bi-specific antibodies on the LNP surface, they were able to achieve mRNA expression in tumor tissues with an ∼8-fold increase compared to untargeted modifications *in vivo.*

**Figure 5:**
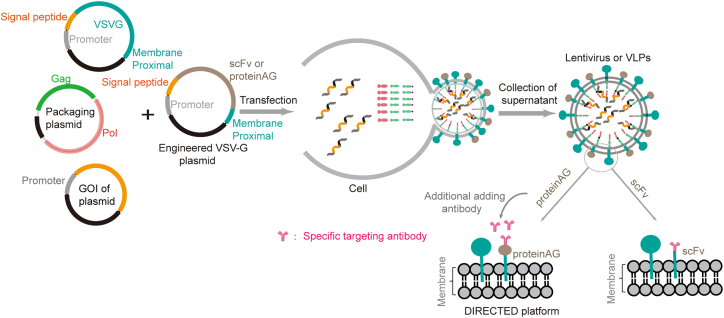
Schematic of VSV-G protein engineering VSV-G was engineered by inserting the coding sequence (proteinAG or scFv) between the secretion signal and the membrane-proximal region. Viruses produced need to co-transfect cells using various ratios of wild-type VSV-G and engineered VSV-G.

The vesicular stomatitis virus G protein (VSV-G) is the most commonly used enveloped protein for VLP RNA delivery systems.^189^ VSV-G interacts with LDL receptor (LDL-R) family members, and the LDL-R family is broadly distributed in different cell types.^189^ Engineered VSV-G has successfully achieved cell-specific delivery. For example, Hamilton et al. fused CD19 scFv (single-chain variable fragment) to the VSV-G N terminus to produce VLPs that packaged Cas9 RNP complexes (CD19-scFv-Cas9-EDV).^44^ Additionally, a HEK293T cell line was created that co-expressed both the B cell ligands CD19^+^ and EGFP (CD19&EGFP HEK293T) to assess the editing efficiency of CD19-scFv-Cas9-EDV in both CD19^+^ and CD19^-^ cells.^44^ In a ∼3:1 mixture of HEK293T and CD19&EGFP HEK293T cells, the CD19-scFv-Cas9-EDVs only induce CD19^+^ and EGFP^+^ cells. Strebinger et al. fused an antibody-binding protein (proteinAG) to the VSV-G N-terminal region to develop a modular platform referred to as DIRECTED (Delivery to Intended Recipient Cells Through Envelope Design) (Figure 5).^189^ The DIRECTED platform separates the fusion and targeting function by using scFv antibodies. It offers a more convenient and easily adaptable way to target different cells or tissues, simply by replacing the targeting antibody (scFv) before recovering the VLPs.

## Clinical trials based on CRISPR-Cas9 mRNA

The CRD-TM-001 (NCT05514249) project involved the *in vivo* delivery of CRISPR-Cas9 targeting the Duchenne muscular dystrophy (*DMD*) gene for the treatment of DMD using the rAAV9 vector.^38^ Unfortunately, it was reported that the first patient experienced acute decompensated and sustained cardiac arrest 6 days after dosing and subsequently succumbed 2 days later.^38^ The cause of death was not clearly explained, but it is possible that treatment with a high dose of rAAV9 led to capillary leakage into the patient’s lungs, resulting in lung damage and a strong immune response, as revealed by postmortem examination. Further analysis of vector biodistribution data showed minimal expression of Cas9 in the liver, suggesting that the cause of death was not directly related to CRISPR-Cas9.^38^ This highlights the importance of delivery vectors as a crucial factor in mediating CRISPR-Cas9-based clinical therapy.

The NTLA-2001 project (NCT04601051) was the first clinical trial utilizing CRISPR-Cas9 mRNA. In this trial, LNP was used to encapsulate sgRNA and Cas9 mRNA targeting the *TTR* gene for the treatment of transthyretin amyloidosis. The phase 1 clinical trial demonstrated the durable knockout of TTR after a single dose, and subsequent serial safety assessments showed only a few mild adverse events.^48^ Five out of six patients showed an increase in D-dimer levels 1 day after *in vivo* administration, but these values returned to the baseline in all patients on day 7. Representative measures of liver function, such as aspartate aminotransferase and alanine aminotransferase levels, remained at a normal level. Dose-dependent pharmacodynamic effects were observed on day 14, indicating reductions from the baseline in the serum TTR protein concentration. Additionally, on day 28, which was when the drug effect had reached its permanent nadir in preclinical studies, the mean reduction from the baseline in serum TTR protein concentration was 52% and 87% in the groups that received a dose of 0.1 and 0.3 mg per kilogram, respectively. Several off-target prediction methods (i.e., Cas-OFFinder, GUIDE-seq, and SITE-seq) identified seven potential off-target loci. Using next-generation sequencing, no evidence of off-target editing (editing ratio ≤ 0.5%) was found for these sites when using NTLA-2001 in a 3-fold dose-response range from 0.007 to 15.5 nM of sgRNA. High doses of NTLA-2001 (i.e., 0.7 and 1.0 mg/kg) were also evaluated in subjects with NYHA class I/II heart failure along with one dose (i.e., 0.7 mg/kg) in subjects with NYHA class III heart failure.^190^ Serum TTR levels were significantly reduced from the baseline in all subjects (mean > 90% at 28 days), and the reduced levels were maintained for 4–6 months. Two of the 12 patients reported transient infusion reaction that resolved without any clinical sequelae.

The VERVE-101 project (NCT05398029) was the first clinical research utilizing LNP-based delivery of mRNA encoding an ABE editor, and it used a single sgRNA targeting *PCSK9* to remove LDL cholesterol for treatment of heterozygous familial hypercholesterolemia.^8^^,^^49^ The VERVE-101 trial, part of the heart-1 clinical trial, has presented results for 10 patients who received an infusion of VERVE-101.^191^ 6/10 patients were administered sub-therapeutic doses (0.1 mg/kg, *n* = 3 and 0.3 mg/kg, *n* = 3), while 4/10 patients received potentially therapeutic doses (0.45 mg/kg, *n* = 3, 0.6 mg/kg, *n* = 1). Two patients showed reductions of 39% and 48% in LDL-C, and PCSK9 levels decreased by 59% and 84% after receiving a 0.45 mg/kg dose. Reduction of 55% in LDL-C lasted up to 180 days, and PCSK9 levels decreased by 47% after receiving a 0.6 mg/kg dose. Serious adverse cardiovascular events, including cardiac arrest, myocardial infarction, and arrhythmias, occurred in two patients with underlying advanced coronary artery disease. The assessment showed that only one of the two adverse events was likely to be related to the treatment. Currently, clinical research based on CRISPR-Cas9 is more focused on *ex vivo* editing, such as *in vitro* modification of T cells or CD34-positive hematopoietic stem cells and hematopoietic stem and progenitor cells, which are then returned to the patient to treat diseases.^192^^,^^193^ Table 1 shows a listing of ongoing *in vivo* CRISPR-Cas9-based clinical studies.

**Table 1:** Ongoing clinical trials studies with CRISPR-Cas9

Name/Clinicaltrials.gov number	Type of Cas9	Target of gene	Diseases	Route of administration	Delivery vectors and cargo of forms	Phase	References
NTLA-2001/NCT04601051	*SpCas9*	*TTR*	ATTR amyloidosis	intravenous	LNP, mRNA	3	Sabnis et al.^194^; Gillmore et al.^48^
VERE101/NCT05398029	ABE7.10	*PCSK9*	LDL cholesterol	intravenous	LNP, mRNA	1	Rothgangl et al.^8^; Horie and Ono^191^
YOLT101/NCT06458010	hpABE5	*PCSK9*	LDL cholesterol	intravenous	LNP, mRNA	1	Wan et al.^195^
ART002/ChiCTR2400093099	SpCas9	*PCSK9*	LDL cholesterol	intravenous	LNP, mRNA	1	Qiu et al.^196^
NTLA2002/NCT05120830	SpCas9	*KLKB1*	hereditary angioedema (HAE)	intravenous	LNP, mRNA	2	Seitzer^197^
YOLT203/NCT06511349	YolCas12F	*HA01*	primary hyperoxaluria type 1(PH1)	intravenous	LNP, mRNA	1	Jiang et al.^198^
BD113/NCT0645537	SpCas9	*MYOC*	primary open-angle glaucoma (POAG)	intravitreous	VLPs, RNP	2	Zhao et al.^199^
EDIT-101/NCT03872479	SaCas9	*CEP290* IVS26	Leber’s congenital amaurosis type 10 (LCA10)	subretinal injection	AAV5, DNA	2	Maeder et al.^5^
EBT101/NCT05144386	SaCas9	*SIVmac239* genome	HIV infection	intravenous	AAV9, DNA	1	Mancuso et al.^200^
CRD-TMH001/NCT05514249	SpCas9	*DMD*	Duchenne muscular dystrophy (DMD)	intravenous	AAV9, DNA	1	Johnson^38^; Lek et al.^38^

Clinicaltrials.gov; search date: 01/October/2025. SpCas9, *Streptococcus pyogenes* Cas9; SaCas9, *Streptococcus aureus* Cas9; hpABE5, an updated version of adenine base editor (ABE) based on ABE8e evolution.

## Trends in CRISPR-Cas9-based gene therapy

The CRISPR-Cas9 system uses sgRNA to direct the Cas9 protein to binding target DNA sequences, after which Cas9 induces double-strand breaks (DSBs).^1^ These DSBs are primarily repaired by two pathways: non-homologous end joining or homology-directed repair pathways. However, CRISPR-Cas9 can induce DSBs at undesired sites, which can lead to chromosome rearrangements, perturbations of essential human gene functions, and subsequent loss of vital cellular functions.^201^^,^^202^

**Figure 6:**
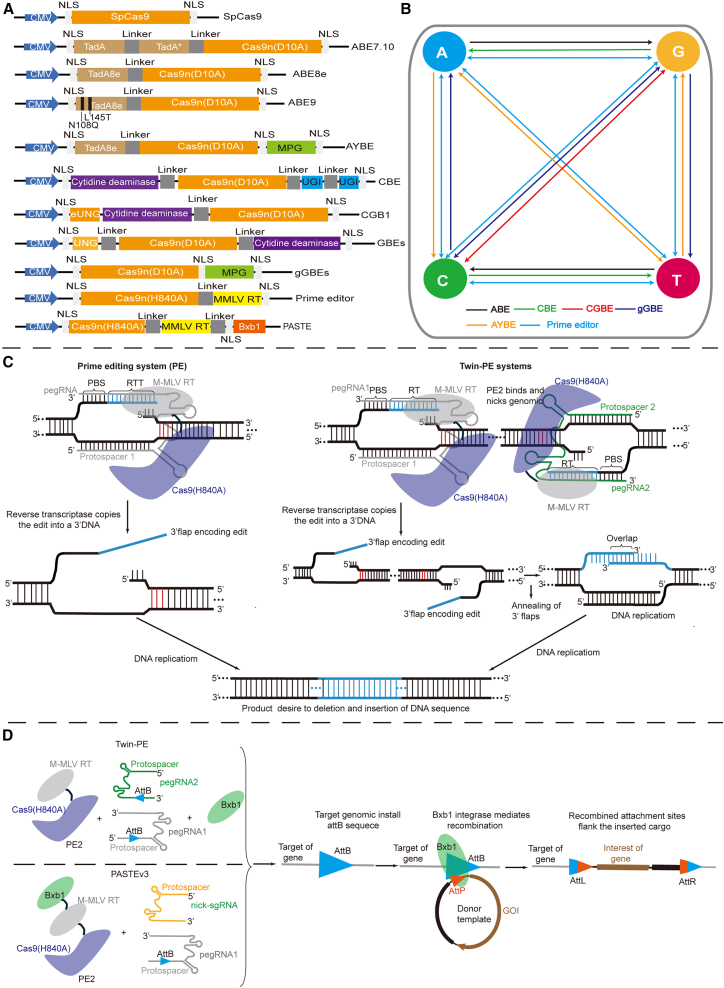
Novel gene editors based on CRISPR-Cas9 development (A) Schematic for the designs of novel editors. eUNG, *Escherichia coli*-derived uracil DNA N-glycosylase; UNG, uracil DNA N-glycosylase; NLS, nuclear localization sequence; MPG, N-methylpurine DNA glycosylase; TadA, adenine deaminases; UGI, uracil glycosylase inhibitor; M-MLV RT, Moloney murine leukemia virus reverse transcriptase. (B) Diagram showing the types of point mutations achieve with BEs and PEs. Arrows indicate the direction of the nucleotide conversion. (C) Schematic of the PE system and TwinPE. PE is achieved via PE2-pegRNA binding and nicking of DNA target sites. The MMLV-RT uses RTT as the template to copy the edit into 3′ DNA from the 3′ flap encoding the edit, then the 3′ flap encoding the edit permanently copies the edited sequence into the non-edited strand by an endogenous cellular pathway.^212^^,^^213^ The PE system in human cells mediates single-nucleotide mutations and insertions of up to ∼40 bp and deletion of up to ∼80 bp. PBS, primer binding site; RTT, reverse transcription template.^212^^,^^213^ The TwinPE system typically contains two protospacer sequences on opposite DNA strands. PE2-pegRNA complexes generate a single-stranded nick on opposing strands of DNA and reverse transcribe the pegRNA-encoded template containing the desired insertion sequence, while insertion sequence does not have to be full length and it can be partially overlapping.^214^ The Twin-PE system in human cells mediates insertions of up to ∼250 bp and deletions of up to ∼10k bp.^213^ (D) Schematic of programmable gene large DNA sequence insertion with Twin-PE and PASTE. Both systems are based on utilizing PE2-pegRNA complexes to install *attB* sequences at a target gene and then use the Bxb1 recombinase to mediate the integration of the donor DNA into the sites.^214^^,^^215^ Both systems mediate targeted integration of a gene size over 5k bp.

However, novel gene editors developed based on Cas9n (Cas9 nickase [D10A] or Cas9 nickase [H840A]) offer a way to circumvent the induction of serious DSBs. These Cas9n-based editors enable direct conversion of one DNA base to another at the programmable target location, creating only single-strand breaks.^203^^,^^204^ Various novel editors have already been developed based on Cas9n, including base editors tools (BEs)^205^^,^^206^^,^^207^^,^^208^^,^^209^^,^^210^^,^^211^ and search-and-replace genome editing technology, named PE tools^212^ (Figures 6A–6C). For instance, cytosine base editors^205^ catalyze the conversion of C•G base pairs to T•A base pairs while ABEs^206^ catalyze the conversion of A•T to G•C mutations. Moreover, C-to-G BEs^207^^,^^208^^,^^209^ induce C-to-G base transversion, and adenine transversion base editing^210^ induces C-to-G and A-to-Y (Y =C, T) transversion. These BE approaches rely on deamination of C and A as the critical step to produce deoxyuridine (U) and deoxyinosine (I) intermediates, respectively. Recently, Tong et al. developed a deaminase-free glycosylase-based guanine (G) BE for catalyzing the conversion of G to Y (Y = C, T) by fusing Cas9n with an engineered N-methylpurine DNA glycosylase protein.^211^

The novel BEs are capable of mediating a high level of editing activity, but their broad editing window, usually editing window of 4–8 nucleotides, increases the risk of bystander effects that induce missense or stop codon mutations.^216^ Chen et al. used protein engineering techniques to develop a novel ABE, namely, ABE9, based on ABE8e.^217^ The ABE9 refined the editing window to 1–2 nucleotides while maintaining a high editing activity. This change represents a significant step in enhancing the precision and specificity of base editing technologies, thus making ABE9 more suitable for precise genome editing applications. Consequently, apart from broadening their targeting scope and enhancing their editing activity, paramount attention should be given to the augmentation of editing specificity, such as refining the editing window.

Although the combinations of all BEs can mediate all 12 possible base-to-base conversions, it is difficult for single BE to repair all 12 different types of point mutations (Figure 6B). Anzalone et al. developed PE editor that not only mediates all 12 possible base-to-base conversions but also mediates small insertions and deletions.^212^ This PE is based on Cas9n fused to an engineered reverse transcriptase (MMLV-RT) and programmed with a prime editing guide sgRNA (pegRNA) that directly writes new genetic information into specified DNA sites (Figure 6C).^212^ While the developed PEs have great potential, they are still challenged by low editing activity, and many groups have reported novel PE effector proteins or pegRNAs that improve editing efficiency. For example, updated version has been developed by incorporating an additional high-mobility group peptide, a hRad51 DNA-binding domain, and through optimized PE effector proteins, NLS sequences, and linkers to develop novel PE editor, named CMP-PE-V1,^218^ hyPE2,^219^ and PEmax,^219^ respectively. Additionally, engineering to pegRNA to improve the PE editing effect, for example, novel epegRNA is by incorporating pseudoknots (evopreQ_1_)^220^ at the 3′ end that increase pegRNA stability, therefore improving editing efficiency.

However, PEs can only induce small insertions (less than 50 nucleotides) and cannot insert or replace large sequences of DNA. Anzalone et al. described a twin prime editing (TwinPE) tool, which is based on a PE that uses two pegRNAs to induce the programmable replacement or excision of DNA sequences at endogenous human genomic sites^214^ (Figure 6C). The TwinPE enabled a 16% insertion efficiency for the 108-bp sequences, which was a 20-fold improvement compared to the original PE approaches. On the other hand, serine integrases (e.g., Bxb1) typically insert sequences containing an *attP* attachment site into a target containing the related *attB* attachment site.^221^ To this end, Anzalone et al. explored a combination of TwinPE and Bxb1 for mediating the attachment site insertion by using a single transfection plasmid encoding PE2 along with both pegRNAs, a plasmid expressing Bxb1 and *attP*-containing DNA donor plasmid (triple-plasmid systems) (Figure 6D). When combined with Bxb1 serine recombinase, the TwinPE enabled targeted integration of gene-sized DNA plasmids (>5,000 bp). Furthermore, Yarnall et al. also developed programmable addition via site-specific targeting elements editing tools, named PASTE, based on Bxb1 serine integrases and PE (Figure 6D).^215^ PASTE successfully allowed programmable integration of cargoes up to ∼36 kb in a single delivery reaction with a high integration efficiency of up to ∼50%–60% in cells. This was achieved by fusing Cas9n, reverse transcriptase, and serine integrases (Bxb1) in one plasmid, engineering the *attB* sequences to pegRNA (termed atgRNA), and placing *attP*-containing DNA donor sites in another plasmid.^215^

PE tools do not require DSBs and can make virtually any substitution, insertion, or deletion and thus have great potential compared to BEs. However, delivery large size sequences (>6 kb) impose challenges, and ineffective editing efficiency remains a major constraint for the *in vivo* use of PE tools. Thus, development of novel delivery vectors and improving the editing activity are the key factors to realizing the potential of PE tools and its derived editing tools for treating various diseases. Additionally, CRISPR-Cas9, BEs, and PEs are overly dependent on sgRNA specificity and enzymatic function. When targeting distinct mutation sites within the same gene, there is an unavoidable need to redesign the systems for each case, which is cumbersome and expensive from a drug development point of view. Nonetheless, the length fragmet integration tenchnology (e.g., Twin PE and PASTE), by installing a Bxb1 sequence at genomic safe harbor loci, means that just one cDNA acts as a donor and a single sgRNA can respond to all mutations in this gene.^222^

Lastly, current engineering strategies for CRISPR-Cas systems tend to focus on two key areas. One is the miniaturization of CRISPR-Cas systems to achieve single AAV packaging and delivery. As is well known, the restrictive cargo size (∼4.7 kb) of AAV presents an obstacle for packaging CRISPR-Cas9 systems in a single AAV vector. In search of smaller Cas effectors for efficient delivery by a single AAV, through protein evolutionary tracing, novel mini-Cas effectors were found, such as Cas12 family proteins ranging in size from approximately 400 to 1,400 amino acids,^223^ *Staphylococcus aureus* (SaCas9) protein with a size of 1,053 amino acids,^224^ TnpB Cas effector proteins with a size of 408 amino acids,^225^ etc. On the other hand, to avoid DNA integration into the host genome, choice to deliver gene-editing agents in RNA or RNP cargo forms is an ideal method. Delivery platforms such as VLP, mLP, SEND, and LNP have emerged as potentially promising vehicles for delivering gene-editing agents.

## Conclusions

CRISPR-Cas9 has brought about revolutionary changes in the field of gene therapy, and with CRISPR-Cas9 systems, we can conveniently and directly manipulate genes in the genome to treat hereditary diseases. However, the potential of off-target effects remains a key obstacle for clinical applications. Off-target events are usually high when CRISPR-Cas9 is continuously expressed. To compare differences in off-target effects, various forms of CRISPR-Cas9 have been studied. Among these, mRNA forms of CRISPR-Cas9-based gene therapy are considered the most promising forms for *in vivo* administration. Unlike immunostimulant therapeutics that require minimal protein amounts, gene therapeutics utilizing CRISPR-Cas9 mRNA require highly efficient translation to reach therapeutic thresholds. However, the instability and short half-life of mRNA, as well as its potential immunogenicity, inevitably lead to inefficient translation of Cas9 mRNA.

Chemically modified or codon-optimized RNA, circRNA, saRNA, and other modified forms have been developed to address these concerns. The novel RNA has improved RNA stability and immunogenicity. However, the novel RNAs also face challenges. For example, circRNA has shown inefficient looping formation rates when the mRNA size exceeds 5,000 bp.^120^ A typical CRISPR-Cas9 system has a size of about 5,000 bp, thus using circular Cas9 mRNA is indeed a challenge because of its large size. Additionally, saRNA is over 10,000 bp when inserted into a ∼2,000 bp sequence, which imposes a challenge for *in vitro* transcriptions because increased transcript lengths have the propensity to result in the generation of truncated mRNA fragments.^226^ Furthermore, modified ribonucleotides incorporated into *in vitro*-transcribed mRNA may cause +1 ribosomal frameshifts and an increase in missense rating.^227^ However, the mechanism by which the modified nucleotides lead to missense or +1 frameshifting and how this affects mRNA translation fidelity remain unclear. Therefore, it is necessary to investigate the impact of incorporating modified ribonucleotides into *in vitro*-transcribed mRNA, particularly concerning the fidelity of translation and potential risks when used in the CRISPR-Cas9 system.

Delivery vectors with high efficiency and target specificity can improve CRISPR-Cas9 editing activity and improve *in vivo* safety. In case of LNPs as a delivery vector for CRISPR-Cas9 systems, modifying the surface of LNPs with targeting moieties, such as antibodies or peptides, and incorporating specific excipients in the LNP formulation, such as SORT molecule or amide-containing lipidosis, can significantly enhance both editing activity and tissue-specific editing.^23^^,^^24^^,^^25^ On the other hand, VLPs and EV-based systems are promising approaches to deliver CRISPR-Cas9 mRNA in the foreseeable future. However, there is a need to assess their immunogenicity and to confirm the maximum or optimal RNA length for specific targeting that VLPs and EVs can deliver. In any case, the rapidly advancing mRNA technology and delivery platforms hold significant promise for gene therapy. We envision that mRNA-driven CRISPR-Cas9-based gene therapy will offer potential new treatment approaches for diseases currently lacking effective therapies, thus addressing unmet clinical needs by precisely targeting and correcting disease-causing genes.

## Acknowledgments

This work was supported by from the 10.13039/501100001809National Natural Science Foundation of China (grant no. General Project, 82372145, 82201287, 82225014, and 82171148), the Joint Fund of the Zhejiang Provincial Natural Science Foundation (LBD24H180001), the 10.13039/501100003395Shanghai Municipal Education Commission (2023ZKZD12), the 10.13039/501100003399Science and Technology Commission of Shanghai Municipality (23J31900100), the Xuhui District Hospital-Land Cooperation Program of Shanghai (23XHYD-05), the 10.13039/501100004731Zhejiang Provincial Natural Science Foundation of China (BD24H180004), the Project (347897), Solution for Health Profile (336355), and InFLAMES Flagship (337531) grants from the 10.13039/501100002341Research Council of Finland. This study is part of the activities of the Åbo Academy University Foundation (SÅA) funded by Center of Excellence in Research “Materials-driven solutions for combating antimicrobial resistance (MADNESS). S.W. (CSC:202307960008) was sponsored by the 10.13039/501100004543China Scholarship Council.

## Author contributions

H.Z. and Y.S. organized and designed the manuscript; S.W. and X.X. performed the literature search, wrote the manuscript, and created the figures; T.V., H.Z., and Y.S. revised the manuscript. All authors read and approved the final manuscript.

## Declaration of interests

The authors declare no competing interests.
